# Characteristics of Hospitalized and Nonhospitalized Patients in a Nationwide Outbreak of E-cigarette, or Vaping, Product Use–Associated Lung Injury — United States, November 2019

**DOI:** 10.15585/mmwr.mm6846e1

**Published:** 2019-11-22

**Authors:** Kevin Chatham-Stephens, Katherine Roguski, Yunho Jang, Pyone Cho, Tara C. Jatlaoui, Sarah Kabbani, Emily Glidden, Emily N. Ussery, Katrina F. Trivers, Mary E. Evans, Brian A. King, Dale A. Rose, Christopher M. Jones, Grant Baldwin, Lisa J. Delaney, Peter Briss, Matthew D. Ritchey, Lung Injury Response Epidemiology/Surveillance Task Force, Lung Injury Response Clinical Task Force, Lung Injury Response Epidemiology/Surveillance Task Force, Kayla Anderson, Francis B. Annor, Sharyn E. Brown, Angela D. Coulliette-Salmond, Kristen N. Cowan, Angela Dunn, Aaron Fleishauer, Jennifer Freed, Macarena C. Garcia, Janet Hamilton, Donald Hayes, Michelle M. Hughes, Mia Israel, Anne Kimball, Hannah Kisselburgh, Jennifer Layden, Akaki Lekiachvili, Matthew Lozier, Ruth Lynfield, Jonathan Meiman, Erin D. Moritz, Rashid Njai, Jeffrey Ratto, Sarah L. Shafer, Stephen Soroka, Natalie Sterrett, Kimberly R. Thomas, Bailey Wallace, Angela K. Werner, Jason Wilken, Lauren B. Zapata, Lung Injury Response Clinical Task Force, Susan Hocevar Adkins, Emily A. Kiernan, Ram Koppaka, Emily H. Koumans, Mark Layer, Jaswinder Legha, Michelle Montandon, Sarah Reagan-Steiner, David A. Siegel, David N. Weissman, Jennifer L. Wiltz

**Affiliations:** ^1^National Center on Birth Defects and Developmental Disabilities, CDC; ^2^National Center for Emerging and Zoonotic Infectious Diseases, CDC; ^3^National Center for Immunization and Respiratory Disease, CDC; ^4^National Center for Chronic Disease Prevention and Health Promotion, CDC; ^5^National Center for Environmental Health, CDC; ^6^National Center for Injury Prevention and Control, CDC; ^7^National Institute for Occupational Safety and Health, CDC.; National Center on Birth Defects and Developmental Disabilities, CDC; National Center for Injury Prevention and Control, CDC; National Center for Chronic Disease Prevention and Health Promotion, CDC; National Center for Emerging and Zoonotic Infectious Diseases, CDC; National Center for Environmental Health, CDC; Utah Department of Health; North Carolina Department of Health and Human Services; Agency for Toxic Substances and Disease Registry; Center for Surveillance; Epidemiology, and Laboratory Services, CDC; Council of State and Territorial Epidemiologists; National Center for Chronic Disease Prevention and Health Promotion, CDC; National Center on Birth Defects and Developmental Disabilities, CDC; Council of State and Territorial Epidemiologists; Epidemic Intelligence Service, National Center for HIV/AIDS, Viral Hepatitis, STD, and TB Prevention, CDC; National Center for Emerging and Zoonotic Infectious Diseases, CDC; Illinois Department of Public Health; National Center for Chronic Disease Prevention and Health Promotion, CDC; National Center for Emerging and Zoonotic Infectious Diseases, CDC; Minnesota Department of Health; Wisconsin Department of Health; National Center for Environmental Health, CDC; National Center for Chronic Disease Prevention and Health Promotion, CDC; Center for Surveillance, Epidemiology, and Laboratory Services, CDC; National Center for Immunization and Respiratory Diseases, CDC; National Center for Emerging and Zoonotic Infectious Diseases, CDC; National Center for Immunization and Respiratory Diseases, CDC; Center for Surveillance, Epidemiology, and Laboratory Services, CDC; National Center on Birth Defects and Developmental Disabilities, CDC; National Center for Environmental Health, CDC; California Department of Public Health; National Center for Chronic Disease Prevention and Health Promotion, CDC.; National Center for HIV/AIDS, Viral Hepatitis, STD, and TB Prevention, CDC; Agency for Toxic Substances and Disease Registry; National Center for Immunization and Respiratory Diseases, CDC; National Center for Chronic Disease Prevention and Health Promotion, CDC; National Center for Environmental Health, CDC; National Center for Injury Prevention and Control, CDC; Center for Global Health, CDC; National Center for Emerging and Zoonotic Infectious Diseases, CDC; National Center for Chronic Disease Prevention and Health Promotion, CDC; National Institute for Occupational Safety and Health, CDC; National Center for Chronic Disease Prevention and Health Promotion, CDC.

CDC, the Food and Drug Administration (FDA), state and local health departments, and public health and clinical stakeholders are investigating a nationwide outbreak of e-cigarette, or vaping, product use–associated lung injury (EVALI) (*1*). As of November 13, 2019, 49 states, the District of Columbia, and two U.S. territories (Puerto Rico and U.S. Virgin Islands) have reported 2,172 EVALI cases to CDC, including 42 (1.9%) EVALI-associated deaths. To inform EVALI surveillance, including during the 2019–20 influenza season, case report information supplied by states for hospitalized and nonhospitalized patients with EVALI were analyzed using data collected as of November 5, 2019. Among 2,016 EVALI patients with available data on hospitalization status, 1,906 (95%) were hospitalized, and 110 (5%) were not hospitalized. Demographic characteristics of hospitalized and nonhospitalized patients were similar; most were male (68% of hospitalized versus 65% of nonhospitalized patients), and most were aged <35 years (78% of hospitalized versus 74% of nonhospitalized patients). These patients also reported similar use of tetrahydrocannabinol (THC)-containing products (83% of hospitalized versus 84% of nonhospitalized patients). Given the similarity between hospitalized and nonhospitalized EVALI patients, the potential for large numbers of respiratory infections during the emerging 2019–20 influenza season, and the potential difficulty in distinguishing EVALI from respiratory infections, CDC will no longer collect national data on nonhospitalized EVALI patients. Further collection of data on nonhospitalized patients will be at the discretion of individual state, local, and territorial health departments. Candidates for outpatient management of EVALI should have normal oxygen saturation (≥95% while breathing room air), no respiratory distress, no comorbidities that might compromise pulmonary reserve, reliable access to care, strong social support systems, and should be able to ensure follow-up within 24–48 hours of initial evaluation and to seek medical care promptly if respiratory symptoms worsen. Health care providers should emphasize the importance of annual influenza vaccination for all persons aged ≥6 months, including persons who use e-cigarette, or vaping, products (*2*,*3*).

State health departments, the Council of State and Territorial Epidemiologists Vaping Associated Pulmonary Injury Epidemiology Task Force, and CDC developed and disseminated surveillance case definitions and data collection tools (i.e., patient interview and medical record abstraction forms) to monitor and track cases beginning in August 2019.* Some states are using these tools, whereas others elected to use state-specific tools. States and jurisdictions routinely report the number of confirmed and probable EVALI cases to CDC on a voluntary basis and, when available, include data from patient interviews and medical record abstractions. Some states have restricted case finding to hospitalized patients. Proxies (e.g., spouses or parents) were interviewed if patients were too ill or if they had died. Most states and jurisdictions report the number of cases to CDC as case status is determined; however, completing and submitting information from interviews and medical record abstraction can take up to several weeks.

This report provides updated data on patient demographic characteristics and substances used in e-cigarette, or vaping, products among hospitalized and nonhospitalized patients, as well as clinical characteristics observed among nonhospitalized patients, according to cases reported to CDC with available interview data, medical record abstraction data, or both as of November 5, 2019. Nonhospitalized EVALI patients were defined as those receiving care in an outpatient clinic, urgent care, or emergency department without report of hospitalization. Demographic and product use characteristics were compared across groups using the chi-square test, and the median ages of patients were compared using the Wilcoxon rank-sum test. SAS statistical software (version 9.4; SAS Institute) was used for the analysis.

Among 2,016 EVALI patients with available data on hospitalization status, 1,906 (95%) were hospitalized, and 110 (5%) were not hospitalized (Table 1). The nonhospitalized patients were reported from 27 states. Demographic characteristics of hospitalized and nonhospitalized patients were similar; most were male (68%; [1,228 of 1,797] of hospitalized versus 65% [70 of 108] of nonhospitalized patients; p = 0.4) and non-Hispanic white (79% [830 of 1,048] of hospitalized versus 82% [46 of 56] of nonhospitalized patients; p = 0.5). A similar age distribution was observed: 78% (1,395 of 1,800) of hospitalized and 74% (78 of 106) of nonhospitalized patients were aged <35 years (p = 0.3), and median age was 24 years for both hospitalized and nonhospitalized patients (p = 0.9). A higher percentage of hospitalized (55%; 1,039 of 1,896) patients compared with nonhospitalized (12%; 13 of 110) were classified with confirmed cases rather than probable cases (p<0.01). Hospitalized and nonhospitalized patients reported similar use of THC-containing products (83% [932 of 1,122] versus 84% [52 of 62], respectively; p = 0.9) and nicotine-containing products (60% [678 of 1,122] versus 73% [45 of 62], respectively; p = 0.06).

**TABLE 1:** Demographic and e-cigarette, or vaping, product use characteristics among patients with e-cigarette, or vaping, product use–associated lung injury (EVALI) reported to CDC, by hospitalization status — United States, August–November 2019*

Characteristic	No./Total no. (%)^†^	Hospitalized no./Total no. (%)^†^	Nonhospitalized no./Total no. (%)^†^	P-value^§^
**Sex**				
Male	1,298/1,905 (68)	1,228/1,797 (68)	70/108 (65)	0.4
Female	607/1,905 (32)	569/1,797 (32)	38/108 (35)	
**Median age, yrs (range)**	24 (13–78)	24 (13–78)	24 (15–71)	0.9
**Age group (yrs)**				
13–17	293/1,906 (15)	275/1,800 (15)	18/106 (17)	0.3
18–24	721/1,906 (38)	685/1,800 (38)	36/106 (34)	
25–34	459/1,906 (24)	435/1,800 (24)	24/106 (23)	
35–44	256/1,906 (13)	242/1,800 (13)	14/106 (13)	
45–64	141/1,906 (7)	132/1,800 (7)	9/106 (8)	
≥65	36/1,906 (2)	31/1,800 (2)	5/106 (5)	
**Race/Ethnicity^¶^**				
White	876/1,104 (79)	830/1,048 (79)	46/56 (82)	0.5
Black or African American	45/1,104 (4)	43/1,048 (4)	2/56 (4)	
American Indian or Alaska Native	5/1,104 (0)	4/1,048 (0)	1/56 (2)	
Asian, Native Hawaiian, or other Pacific Islander	19/1,104 (2)	19/1,048 (2)	0/56 (0)	
Other	26/1,104 (2)	24/1,048 (2)	2/56 (4)	
Hispanic	133/1,104 (12)	128/1,048 (12)	5/56 (9)	
**Case status**				
Confirmed	1,052/2,006 (52)	1,039/1,896 (55)	13/110 (12)	<0.001
Probable	954/2,006 (48)	857/1,896 (45)	97/110 (88)	
Substances used in e-cigarette, or vaping, products** ^,††^				
THC-containing product (any use)	984/1,184 (83)	932/1,122 (83)	52/62 (84)	0.9
Nicotine-containing product (any use)	723/1,184 (61)	678/1,122 (60)	45/62 (73)	0.06
Both THC- and nicotine-containing product use	573/1,184 (48)	538/1,122 (48)	35/62 (56)	0.2^§§^
THC-containing product use only	411/1,184 (35)	394/1,122 (35)	17/62 (27)	
Nicotine-containing product use only	150/1,184 (13)	140/1,122 (12)	10/62 (16)	
No THC- or nicotine-containing product use reported	50/1,184 (4)	50/1,122 (4)	0/62 (0)	

**Abbreviation:** THC = tetrahydrocannabinol.

* For cases reported as of November 5, 2019.

^†^ Percentages might not sum to 100% because of rounding.

^§^ To assess for statistically significant differences between the hospitalized and nonhospitalized patients, a chi-square test was performed for comparing categorical data and Wilcoxon rank-sum test for the comparison of the median ages.

^¶^ Whites, blacks or African Americans, American Indians or Alaska Natives, Asians, Native Hawaiians or other Pacific Islanders, and Others were non-Hispanic. Hispanic persons could be of any race.

** Data on both THC- and nicotine-containing product use required to be included.

^††^ In the 3 months preceding symptom onset.

^§§^ Comparison of the mutually exclusive categories of “Both THC- and nicotine-containing product use,” “THC-containing product use only,” “Nicotine-containing product use only,” and “No THC- or nicotine-containing product use reported.”

According to medical chart abstraction data reported to CDC on nonhospitalized EVALI patients’ initial outpatient medical visit, 85% (47 of 55) experienced respiratory symptoms (e.g., cough, chest pain, and shortness of breath), 57% (27 of 47) had gastrointestinal symptoms (e.g., abdominal pain, nausea, vomiting, and diarrhea), and 76% (41 of 54) had constitutional symptoms (e.g., fever, chills, and weight loss) (Table 2). Very few patients reported only one symptom type (e.g., 9% [four of 47] reported having only respiratory symptoms). Initial oxygen saturation <95% (while breathing room air) was reported among 30% (eight of 27) of patients and tachycardia among 40% (10 of 25); no patients had tachypnea. Twenty-one (81%) of 26 patients with available data were reportedly prescribed corticosteroids. Among 34 patients with results reported for initial chest radiographs (CXR), 28 (82%) had abnormal findings, and 76% (19 of 25) had bilateral findings; three cases had missing information and were excluded. All 28 patients with results reported for chest computed tomography (CT) scans had abnormal findings, including 27 (96%) with bilateral findings. Six of 16 (38%) patients with information on both a CXR and chest CT had an initial normal CXR but abnormal chest CT; 10 (63%) had both an abnormal CXR and chest CT.

**TABLE 2:** Clinical characteristics among nonhospitalized patients with e-cigarette, or vaping, product use–associated lung injury (EVALI) reported to CDC — United States, August–November 2019*

Characteristic	No./Total no. (%)^†^
**Symptoms reported**	
Any respiratory	47/55 (85)
Any gastrointestinal	27/47 (57)
Any constitutional	41/54 (76)
**Among cases with complete symptom information**	
Respiratory symptoms only^§^	4/47 (9)
Gastrointestinal symptoms only^¶^	0/47 (0)
Constitutional symptoms only**	1/47 (2)
**Vital signs on initial presentation**	
Oxygen saturation <95% while breathing room air	8/27 (30)
Tachycardia (heart rate >100 beats/min)	10/25 (40)
Tachypnea (respiratory rate >20 breaths/min)	0/10 (0)
**Corticosteroids prescribed**	21/26 (81)
**Initial radiographic findings**	
Abnormal chest radiograph	28/34 (82)
Bilateral findings^††^	19/25 (76)
Abnormal chest CT	28/28 (100)
Bilateral findings	27/28 (96)
**Among cases with both chest radiograph and chest CT findings reported^§§^**	
Chest radiograph normal but chest CT abnormal	6/16 (38)
Chest radiograph abnormal but chest CT normal	0/16 (0)
Both abnormal	10/16 (63)

**Abbreviation:** CT = computed tomography.

* For cases reported as of November 5, 2019.

^†^ Percentages might not sum to 100% because of rounding.

^§^ Self-reported symptoms (e.g., cough, chest pain, and shortness of breath).

^¶^ Self-reported symptoms (e.g., abdominal pain, nausea, vomiting, and diarrhea).

** Self-reported symptoms (e.g., fever, chills, and weight loss).

^††^ Three cases had missing chest radiograph information on unilateral versus bilateral findings and were excluded from this calculation.

^§§^ Dates of chest radiographs and CT scans were not consistently reported, so it is unknown whether they were performed on the same or subsequent days, which could explain, in part, why the findings for the imaging tests were inconsistent among some patients.

## Discussion

Available data suggest that nonhospitalized EVALI patients have similar demographic and product use characteristics as do hospitalized EVALI patients. In anticipation of increasing incidence of influenza and other respiratory infections during the winter, CDC engaged with state health departments and clinical partners to assess the value of continuing to report EVALI patients who are not hospitalized. EVALI is a diagnosis of exclusion because, at present, no specific test or marker exists for its diagnosis, and evaluation should be guided by clinical judgment. Because patients with EVALI can have symptoms similar to those associated with influenza or other respiratory infections (e.g., fever, cough, headache, myalgias, or fatigue), it might be difficult to differentiate EVALI from influenza or community-acquired pneumonia on initial assessment, and EVALI might co-occur with respiratory infections. Further, continued case finding and case reporting of patients with EVALI who are treated in the outpatient setting will likely impose a significant burden on health systems and health departments during the emerging 2019–20 influenza season. Given this burden, the demographic and clinical findings from this report suggest that data from these outpatient EVALI patients likely will not provide sufficient additional evidence to the continuing investigation. Thus, CDC is no longer requesting national data on outpatient EVALI patients. Further collection of data on nonhospitalized patients will be at the discretion of individual state, local, and territorial health departments.

The findings in this report are subject to at least four limitations. First, data on substances used in e-cigarette, or vaping, products were self-reported or reported by proxies and might be subject to recall bias or social desirability bias. Therefore, underreporting might have occurred. Second, these data might be subject to misclassification of substance use for multiple reasons. Patients might not know the content of the e-cigarette, or vaping, products they used, and methods used to collect data regarding substance use varied across state. Third, data on some variables were missing for many patients, including where nonhospitalized patients received care (e.g., outpatient clinic, urgent care, or emergency department), and conclusions derived from these data might not be generalizable to the entire patient population. Finally, some states have restricted case finding to hospitalized patients, thus these data likely underestimate the actual number of nonhospitalized EVALI patients.

Candidates for outpatient management of EVALI should have normal oxygen saturation (≥95% while breathing room air), no respiratory distress, no comorbidities that might compromise pulmonary reserve, reliable access to care, strong social support systems and should be able to ensure follow-up within 24–48 hours of initial evaluation and to seek medical care promptly if respiratory symptoms worsen; in some cases, patients who initially had mild symptoms experienced a rapid worsening of symptoms within 48 hours (*2*,*3*). Health care providers should consider obtaining a chest CT scan if patients have an initial normal CXR but have symptoms and an exposure history suggestive of EVALI. Further, health care providers should consider use of antimicrobials, including antivirals, in accordance with established guidelines. Use of corticosteroids for the treatment of EVALI in the outpatient setting should be considered with caution because it might worsen respiratory infections. Health care providers should emphasize the importance of annual influenza vaccination for all persons aged ≥6 months, including persons who use e-cigarette, or vaping, products (*2*,*3*).

Recent CDC laboratory testing has detected vitamin E acetate in bronchoalveolar lavage fluid samples from a convenience sample of 29 patients with EVALI (*4*). These findings provide direct evidence of vitamin E acetate at the primary site of injury within the lungs. However, evidence is not yet sufficient to rule out other chemicals of potential concern contributing to EVALI. Many different substances and product sources are still under investigation, and there might be more than one cause of this outbreak. Therefore, since the specific cause or causes of EVALI are not yet known, the only way for persons to assure that they are not at risk is to consider refraining from use of all e-cigarette, or vaping, products while this investigation continues. In particular, because most patients with EVALI report using THC-containing products before the onset of symptoms, CDC recommends that persons not use e-cigarette, or vaping, products that contain THC, especially those acquired from informal sources like friends, family members, or in-person or online dealers. Persons should not modify or add any substances to e-cigarette, or vaping, products that are not intended by the manufacturer; these include but are not limited to vitamin E acetate and other cutting agents and additives obtained from informal sources or purchased through retail establishments. Irrespective of the ongoing investigation, e-cigarette, or vaping, products should never be used by youths, young adults, or women who are pregnant. Moreover, persons who do not currently use tobacco products should not start using e-cigarette, or vaping, products (*2*). Adults using e-cigarette, or vaping, products to quit smoking should not return to smoking cigarettes; they should weigh all risks and benefits and consider using FDA-approved cessation medications.^†^ Adults who continue to use e-cigarette, or vaping, products should carefully monitor themselves for symptoms and see a health care provider immediately if they develop symptoms like those reported in this outbreak.

**Summary**

**What is already known about this topic?**

A total of 2,172 e-cigarette, or vaping, product use–associated lung injury (EVALI) cases have been reported in the nationwide outbreak as of November 13, 2019; most patients reported using tetrahydrocannabinol-containing products in the 3 months before symptom onset.

**What is added by this report?**

As of November 5, 2019, 5% of EVALI patients were not hospitalized. Hospitalized and nonhospitalized patients had similar demographic and product use characteristics.

**What are the implications for public health practice?**

CDC will no longer collect national data on nonhospitalized EVALI cases. Further collection of data on nonhospitalized patients will be at the discretion of individual health departments. Clinical criteria are available to identify candidates for outpatient management of EVALI. Influenza vaccination should be considered for all persons who use e-cigarette, or vaping, products.

## Footnotes

* https://www.cdc.gov/tobacco/basic_information/e-cigarettes/assets/2019-Lung-Injury-Surveillance-Case-Definition-508.pdf.

† https://www.cdc.gov/tobacco/campaign/tips/quit-smoking/index.html?s_cid.
